# Frequency of Bacterial Agents Isolated From Patients With Chronic Sinusitis in Northern Iran

**DOI:** 10.5539/gjhs.v8n5p239

**Published:** 2015-11-17

**Authors:** Rostam Pourmousa, Roksana Dadashzadeh, Fatemeh Ahangarkani, Mohammad Sadegh Rezai

**Affiliations:** 1 Mazandaran Pediatric Infectious Diseases Research Center, Mazandaran University of Medical Science, Sari, Iran; 2 Antimicrobial Resistance Research Center, Department of Infectious Diseases, Mazandaran University of Medical Sciences, Sari, Iran

**Keywords:** chronic sinusitis, endoscopy, bacteria agent

## Abstract

**Background:**

Sinusitis is a disease with significant health problems. Diagnosis of sinusitis is clinical. The golden standard for detection of microorganisms that cause sinusitis is the culture of sinus drainage discharge.

**Objectives:**

Due to the high prevalence of sinusitis in Iran, especially in Mazandaran province, in this study, bacteriological survey of patients with chronic sinusitis were done in order to help physicians in choosing better antibiotics for the empiric therapy of sinusitis.

**Methods:**

This was a descriptive study. The population of the study consisted of 100 patients with chronic sinusitis caused by bacteria admitted to the Avicenna teaching hospital. Sampling for bacterial culture was performed by the endoscopy method from middle meatus (a curved anteroposterior passage in each nasal cavity that is situated below the middle nasal concha and extends along the entire superior border of the inferior nasal concha) and the opening of the maxillary sinus. Also sampling of nasal cavity was performed to determine the microbial flora. Identification of the bacteria causing chronic sinusitis was performed according to the standard microbiological procedures. Antimicrobial susceptibility testing method, the disk diffusion (Kirby-Bauer) was performed according to the CLSI (Clinical and Laboratory Standards Institute) standards. Data were analyzed using SPSS17 software. Also Fisher exact test and descriptive statistics were used to analyze the data.

**Results:**

Among the 100 evaluated patients, 58% were male. The average age was 34.2±1.1. The most common complaint of patients were nasal congestion and post-nasal drip. The most common bacteria found in the nasopharynx were Gram-positive bacillus, coagulase negative Staphylococcus and Staphylococcus aureus with rates of 20%, 16% and 15% respectively. Bacteria isolated from opening sinus were Gram-positive bacillus 24%, *Enterobacter aerogenes* 10%, coagulase negative Staphylococcus 18% and Staphylococcus aureus 19%.

**Conclusions:**

Antibiotic prescription is often empiric in treatment of sinusitis. In our study resistance to some antibiotics such as penicillin subgroups that are used in treatment of chronic sinusitis was high. Due to the fact that the etiology of chronic sinusitis is not clearly understood, the frequency of all the common causative agents of this disease must be determined.

## 1. Introduction

Sinusitis is a disease that causes significant health problems, societal burden, and still has an unknown pathophysiology (Boase et al., 2013). In the US, people with sinusitis spend more than $2 billion annually, and make 1 million physician visits each year for symptomatic palliation. Sinusitis is the most prevalent disease for adults diagnosed in ambulatory medical care and is treated with antibiotics (Passali et al., 2015). Sinusitis is the inflammation of the paranasal sinuses that can occur because of infections, allergic reactions or autoimmune disorders. Inflammation in sinuses can create swelling and excess mucus. This can block nasal breathing and drainage. The inflammation can have more than one cause such as nasal polyps, allergic reactions, deviated nasal septum, trauma to the face, respiratory tract infections, immune system cells, allergies such as hay fever and other medical conditions such as the complications of cystic fibrosis, gastroesophageal reflux and, HIV and other immune system-related diseases may also result in nasal blockage (Rudmik & Soler, 2015; Caspersen et al., 2015). Most cases of sinusitis are viral sinusitis and it takes about 10 days without antibiotic treatment for the patients to improve. Also Sinusitis may be caused by bacterial agents (Anon, 2010). Bacterial sinusitis are divided into 2 groups based on the duration that sinus is involved: if the sinusitis lasts less than 3 weeks, it is called acute sinusitis. Chronic sinusitis is the involvement of the sinuses for more than 3 months with no response to treatment. The bacteria presumed to be involved in chronic sinusitis differ from those involved in acute sinusitis. The bacteria isolated through endoscopy or sinus puncture in patients with chronic sinusitis are *Streptococcus pneumonia*, *Haemophilus influenza*, *Moraxella catarrhalis*, *Staphylococcus aureus*, Coagulase-negative staphylococci, Gram-negative bacteria such as *Pseudomonas aeruginosa*, *Proteus* spp., *Klebsiella* spp., *Enterobacter* spp., *Escherichia coli* and Anaerobic bacteria (*Peptostreptococcus*, *Prevotella*, *Porphyromonas*, *Bacteroides*, *Fusobacterium* spp. (Bhattacharyya & Kepnes, 1999; Power et al., 2005). Diagnosis of sinusitis is based on clinical detection (Rahmati et al., 2014). The goals of treatment for chronic sinusitis are different, depending on whether the source of infection is viral or bacterial. Culture of sinus secretion is the gold standard for the detection of causative microorganisms. The indiscriminate use of antibiotics for sinusitis patients by physicians, including viral and allergic or bacterial sinusitis can create antibiotic-resistant bacteria. On the other hand bacterial epidemiology that causes sinusitis is not well understood in Iran and the treatments are done based on studies performed in other countries. Due to the fact that European and American countries now routinely vaccinate pneumococcal and *Haemophilus influenzae*, epidemiology of bacteria causing sinusitis in these countries is different from Iran (Plosker, Prymula, & Schuerman, 2009). Due to the high prevalence of sinusitis in Iran, especially in Mazandaran province, in this study, the bacteriological survey of patients with chronic sinusitis were done in order to help physicians in choosing better antibiotics for the empiric therapy of sinusitis.

## 2. Material and Methods

### 2.1 Design, Population, Inclusion and Exclusion Criteria

This was a descriptive study approved by the Ethics Committee of Mazandaran university of medical sciences (Code No90-9, Date: August 3, 2011). The population of the study consisted of 100 patients with chronic sinusitis who had the disease symptoms for more than 12 weeks according to the criteria for chronic sinusitis set by the American Academy of Otolaryngology and they were admitted to Avicenna teaching hospital. The inclusion criteria included: patient with chronic sinusitis that were diagnosed by a subspecialist in pediatric infectious diseases and specialist in otolaryngology that over the past month, did not take any antibiotics. Exclusion criteria included: patients that had taken antibiotics over the past month, patients who had anatomical deformity in a way that their middle meatus was not visible, patients who previously underwent sinus surgery, patients who were immunocompromised due to some underlying disease and diabetes.

### 2.2 Sampling

Given the patient’s condition, nasal cavity was washed under general anesthesia or local anesthesia with saline in a hospital operating room. After shrinkage of mucosa and bilateral media with effusion (OME) (0.0001 Vasoconstrictor impregnated pad was set for 10 minutes in nasal cavity), specimens were taken from the affected maxillary sinus and from the middle meatus and nasopharynx by the endoscopy method. Also sampling of nasal cavity to determine the microbial flora was performed. Samples were transported to the microbiology laboratory in Stuart media.

### 2.3 Bacterial Identification

Samples were cultured in blood agar, eosin methylene blue agar and Chocolate agar and incubated at 37°C for 18- 24 hours. The standard microbiological procedures were performed to identify the microorganisms (Collee et al., 1996; Koneman et al., 1997). Bacterial titers exceed 1000 colony forming units per milliliter of mucus (cfu/mL), were considered as a pathogenic bacteria.

### 2.4 Antibiotic Susceptibility Test

Susceptibility of the clinical isolates to routinely used antibiotics which are Ceftizoxime, Ceftriaxone, Ciprofloxacin, Gentamicin, Cotrimoxazol, Amoxicillin, Ampicillin, Amikacin, Cefazolin, Cephalothin, Penicillin G and Vancomycin was determined by the disk diffusion method (Kirby-Bauer) according to the standards (CLSI, 2010).

### 2.5 Statistical Analysis

Data were analyzed using SPSS17 software. Fisher exact test and descriptive statistics were used.

## 3. Results

**Figure 1:**
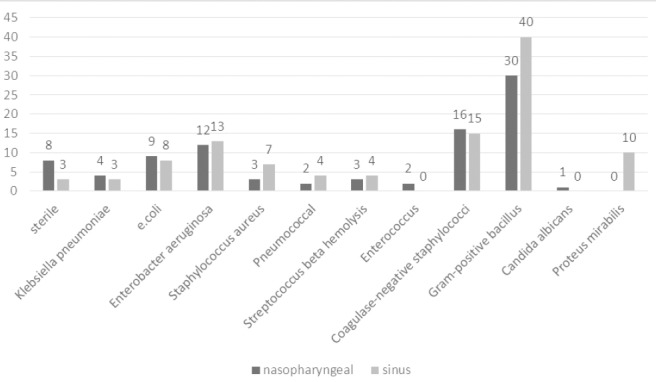
Frequency of microorganisms isolated from nasopharyngeal and sinus

**Figure 2:**
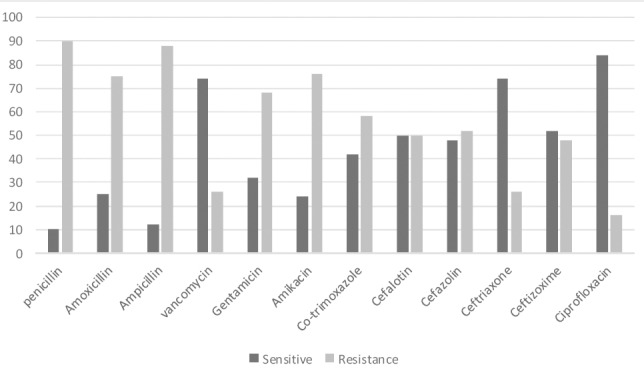
Comparison of the sensitivity and resistance of antibiotic therapy in chronic sinusitis

After reviewing the inclusion criteria, from 135 patients with chronic sinusitis, 100 patients were enrolled in our study (17 patients had used antibiotics over the past month, 11 patients had a history of sinus surgery and 7 patients had anatomical deformity in middle meatus). 58 patients were male (58%) and 42 patients were female (42%) (p=.78). The average age was 34.2±11.1 (the youngest was an 8 year old and the oldest a 68 year old). Demographic Features, symptoms, underlying diseases and the site of sinus involvement in these patients are shown in table 1. Frequency of bacterial agents isolated from nasopharyngeal and sinuses are shown in Figure 1. Also with having in mind the most common bacteria isolated from the sinus, the existence of the same bacteria in the nasopharyngeal of the patients was tested (Table 2). Comparison of the sensitivity and resistance to antibiotic therapy in chronic sinusitis are illustrated in Figure 2.

**Table 1:** Demographic Features, symptoms, underlying diseases and Site of sinus involvement

			P value
Sex	Male	42(42%)	
	female	58(58%)	0.71

Age		34.2±11.1	

Symptoms	Nasal congestion	31(31%)	0.86
	Post nasal drip	24(24%)	
	Sneezing and coughing	21(12%)	
	Chronic Headache	14(14%)	
	Pain around the eyes	9(9%)	
	Itchy and Watery eyes	15(15%)	
	Hoarseness	5 (5%)	
	Bad taste or bad breath	7 (7%)	

Underlying diseases	Asthma and Allergies	31(31%)	0.54
	Diabetes	9(9%)	
	Family history of sinusitis	14(14%)	

Site of sinus involvement	Poly sinuses	38(38%)	0.62
	Ethmoidal sinuses	34(34%)	
	Maxillary sinus	20(20%)	
	Sphenoidal sinuses	6(6%)	
	Frontal sinuses	2(2%)	

**Table 2:** Correlation between sinus and nasopharyngeal bacteria

Sinuses bacteria culture result							

	*staphylococcus aureus*			*staphylococcus* coagulase negative		*Enterobacter aerogenes*	
Nasopharynx bacteria	positive	2(28.5%)	0 (0)	6(40%)	8(71%)	8(61.5%)	0(0)
	negative	5(71.4%)	3(100%)	11(60%)	14(93%)	5(38.4%)	12(100%)
Sensitivity		28.5%		40%		61.5%	
Specificity		100%		93%		100%	
Predictive Value Positive		100%		85.7%		100%	
Predictive Value Negative		37.5%		56%		70.5%	
P value		0.04		0.03		0.001	

## 4. Discussions

Chronic sinusitis as one of the most common diseases of the upper respiratory tract has caught the attention of many researchers and the bacteria involved in it are widely studied (Boase et al., 2013, Witt et al., 2009). The healthy middle meatus and sinus cavity microbiome may be dependent on high constitutive levels of Nitric oxide in the sinus gaseous compartment or other antimicrobial proteins/peptides in sinus fluid (Wilson and Hamilos, 2014). It is important to note that normal flora including *Streptococcus pneumoniae*, *Haemophilus influenzae*, *Staphylococcus* *aureus* and anaerobes such as Bacteroides species, anaerobic gram-positive cocci, and *Fusobacterium* species have been cultured from healthy uninfected sinuses. The presence of these bacteria is only “normal” if the titer of resident flora is low. When bacterial titers exceed 1000 colony forming units per milliliter of mucus (cfu/mL), they are considered pathogenic. Kaspar et al. evaluated the human nose habitats bacterial and they found out that S.aureus was the one found most frequently in association with Staphylococcus epidermidis (Kaspar et al., 2015). In the present study, similar to Gwaltney et al. study, most of the involvement of sinuses were pan sinusitis (Gwaltney, 1996). In consistence with Vogan et al. findings, Sampling in our study was done using swabs to avoid environmental pollution. Vogan showed that sampling by swab from meatus and sinus aspiration does not affect the composition of the bacteria after culture (Vogan et al., 2000). In chronic sinusitis, the accumulation of microbes in culture is a good guide to choose the antibiotics (Desrosiers, 2013). Unlike acute bacterial sinusitis, in chronic sinusitis the combinations of bacteria are more complex and worthy of attention (Feldt, Dion, Weitzel, & Mcmains, n.d.). In our study the most common bacteria isolated from sinuses were Enterobacter aeruginosa (13%), *Staphylococcus* coagulase-negative and gram-positive bacilli (34%). The prevalence of bacteria isolated in our study were in consistence with Hoyt et al. and Yildirim et al. findings (Hoyt, 1992; Yildirim et al., 2004).

In our study, similar to Erkan et al. findings, the rate of anaerobic bacteria was low. Although we have to consider the absence of good facilities for culture of anaerobic bacteria in the north of Iran. Erkan showed that despite long-term antibiotic treatment, bacteriological composition was still intact (Erkan et al., 1996). Also this matter is smilar to Jiang et al. findings on sinusitis patients who were treated with Amoxicillin clavulanate (Jiang et al., n.d.). On the other hand, since the most common complaints and symptoms in patients with sinusitis are post-nasal drip and considering the fact that the drainage of sinus secretions are the same product, It was hypothesized that the culture of nasopharyngeal can be replaced as an alternative for culture of the middle and upper meatus secretions that are difficult for sampling and cause discomfort for patient. So results in the cultivation of the sinuses and nasopharyngeal cultures were compared using Fisher’s exact test. Ilki et al. showed that the correlation between the results of throat and sinus cultures was not sufficient to allow throat culture to be recommended for the bacteriological documentation of sinusitis (Ilki et al., 2005). In our study, the relationship between the results of sinus and nasopharyngeal cultures of three of the most common bacteria involved with chronic sinusitis (coagulase-negative *staphylococcus*, *Staphylococcus aureus*, *Enterobacter aerogenes*) was significant (p=<05).

Biswas et al. indicated that most of the variations in bacterial compositions can be explained by inter-personal differences, rather than sampling location or even disease status (Biswas et al., 2015). Overall, after elimination of the common agents (poly-microbial), in 84% of the cases, nasopharyngeal culture results were consistent with sinus culture results. This finding was in contrast to results of Orobello et al. that there was a significant association only in 45% of cases (Orobello et al., 1991). In our study, positive culture results were reported in 97% of the cases. Positive cultures were included Gram-positive and Gram-negative aerobic bacteria, anaerobic, or a combination of them. In Ragab et al. study Samples taken from the middle meatus were 71% positive culture (Ragab et al., 2007). Germs obtained from sinuses were as follows: Gram-positive bacillus (34%) (We couldn’t culture it in our laboratory due to the absence of necessary facilities), *Enterobacter aerogenes* (13%), coagulase negative *staphylococcus* (15%) and *staphylococcus aureus* (7%). Brook proved that the anaerobic bacteria in chronic sinusitis are important pathogens (Brook, 2002, 2005).

Schlosser et al. showed that the most common agents of chronic sinusitis were bacteria such as coagulase-negative *Staphylococci* and *Staphylococcus aureus*, which is consistent with other studies (Doyle & Woodham, 1991; Biel et al., n.d.; Schlosser et al., 2001). Also in Orobello and colleagues study, the first ranked bacteria of 39 samples taken from the children with sinusitis was coagulase-negative staphylococci (46%) (Orobello et al., 1991). The total number of Gram-negative species in this study was about 24% which is in consistence with Kirtsreesaku et al. findings. Kirtsreesku et al. showed that middle meatus sampling is as a shortcut for evaluation and treatment of maxillary sinusitis (Kirtsreesakul et al., 2005).

Overall, in our study compared to other studies, aerobic bacteria especially Gram-positive bacteria were the most common causes of chronic sinusitis. Also in treatment of chronic sinusitis we should not be unaware of Gram-negative and anaerobic bacteria, and fungi. We showed that most antibiotic resistance among isolated bacteria was to ampicillin (80%) and penicillin G (90%). In many geographical areas, penicillin group is a first-line antibiotic for treatment of sinusitis (Shanmugam et al., 2011). Studying individual species and overall all the bacteria has indicated that antimicrobial resistance is increasing in bacteria especially to ampicillin and penicillin G among Gram positives. Prescription of suitable antibiotics is strongly recommended and physicians need to take the prevalence and antibiotic resistance patterns of the bacteria causing sinusitis into consideration. Also it is necessary to teach patients to avoid inappropriate consumption of antibiotics.
